# Black Tea Source, Production, and Consumption: Assessment of Health Risks of Fluoride Intake in New Zealand

**DOI:** 10.1155/2017/5120504

**Published:** 2017-06-21

**Authors:** Declan T. Waugh, Michael Godfrey, Hardy Limeback, William Potter

**Affiliations:** ^1^EnviroManagement Services, 11 Riverview, Dohertys Rd, Bandon, Co. Cork P72 YF10, Ireland; ^2^Bay of Plenty Environmental Health Clinic, 1416A Cameron Road, Tauranga 3012, New Zealand; ^3^Faculty of Dentistry, University of Toronto, 124 Edward Street, Toronto, ON, Canada M5G 1G6; ^4^Department of Chemistry and Biochemistry, KEH M2225, University of Tulsa, Tulsa, OK, USA

## Abstract

In countries with fluoridation of public water, it is imperative to determine other dietary sources of fluoride intake to reduce the public health risk of chronic exposure. New Zealand has one of the highest per capita consumption rates of black tea internationally and is one of the few countries to artificially fluoridate public water; yet no information is available to consumers on the fluoride levels in tea products. In this study, we determined the contribution of black tea as a source of dietary fluoride intake by measuring the fluoride content in 18 brands of commercially available products in New Zealand. Fluoride concentrations were measured by potentiometric method with a fluoride ion-selective electrode and the contribution of black tea to Adequate Intake (AI) and Tolerable Upper Intake Level (UL) was calculated for a range of consumption scenarios. We examined factors that influence the fluoride content in manufactured tea and tea infusions, as well as temporal changes in fluoride exposure from black tea. We review the international evidence regarding chronic fluoride intake and its association with chronic pain, arthritic disease, and musculoskeletal disorders and provide insights into possible association between fluoride intake and the high prevalence of these disorders in New Zealand.

## 1. Introduction

Internationally, New Zealand (NZ) is one of the highest black tea per capita consumption countries [1]; yet no information is available to consumers on the fluoride (F) levels in black tea products in NZ. It is now recognized that, of all the common foodstuffs, tea produced from the leaves of* Camellia sinensis* has one of the greatest potentials for increasing the daily fluoride (F) intake [2–10]. Tea has long been considered as a useful natural source of F for the prevention of dental caries [11–16] and its anticariogenic effect has been widely demonstrated in a number of human studies [17–23]. Indeed, Onisi et al. (1978) suggested that a solution to the psychological, political, and ethical obstacles to water fluoridation and the use chemical fluorides was recommending tea consumption [24]. Moreover, Onisi et al. (1981) found that drinking a cup of tea daily with a fluoride content of 0.48 mg was more protective of oral health than water fluoridation [19]. A recent large-scale observational study conducted in the United States of America (USA) and England which examined the oral health of adults aged 25 years and over found that mean number of missing teeth was significantly higher in the US than in England [25]. Considering that over 70% of the USA population and <10% of the UK population are provided with fluoridated drinking water, this would appear to suggest that tea consumption is better than water fluoridation for oral health. However, it should be noted that most of the reports suggest that the anticaries effect observed with tea is due primarily to the antibacterial properties of the organic components (polyphenols and tannins) rather than the cariostatic effect of F [19, 26, 27].

The earliest reports identifying tea as a hyperaccumulator of F appear to be those published in the 1930s [28, 29], approximately a decade before the time when fluoridation of public water was initiated in the USA. In 1954, dental researchers cautioned that while fluoridation of communal water supplies was expanding rapidly in the USA in consideration of fluoridation in the UK, it should be recognized that Britons already take in appreciable amounts of F from drinking tea [10]. Early studies by Jackson and Weidmann (1958) reported that tea was a significant contributor to bone F levels in humans. Even in the absence of F in water, there was at least a tenfold increase in bone F levels over time, which was attributed to black tea consumption in England [30]. Similarly, in the Persian Gulf, early studies by Azar et al. (1961) observed that habitual and excessive tea drinking may exaggerate the manifestation of fluorosis [31]. Many individuals drink tea rather than water as their primary means of fluid intake [32, 33]. Most people drink tea as an infusion (adding hot or boiling water); however, in some countries, including India, China, and Egypt, tea is drunk as a decoction (tea and water are boiled together) [34].

It is reported that tea leaves contain 98% of the F of the whole plant [35], with the most accumulated in old leaves [36]. Most of the fluorine accumulated in the leaves is in the form of the F anion [37]. The four types of tea most commonly found in the market are black tea (BT), green tea (GT), oolong tea, and white tea. The difference among them lies in the different processing or, in the case of white tea, different harvesting times. White tea leaves are picked and harvested before they fully open; this is done when the buds are still covered by fine white hair. In the case of white tea and GT, the leaves are steamed quickly (Japanese style) or roasted in pans with dry heat (Chinese type) after harvesting to prevent oxidation of polyphenols. Prior to final drying, the leaves are pressed and rolled, developing their characteristic shape and sizes. After drying, the leaves are sorted into various grades. In the production of BT, the leaves are oxidized (also called “fermentation”) before drying and grading. To accelerate the oxidation process, the leaf size is reduced by rupturing the withered tea leaf using “orthodox rollers” or “crush-tear-curl” (CTC) machines. BT produced using rollers is more suitable for large-leaf-type tea (orthodox), whereas CTC method leads to obtaining small particles suitable for teabags (fanning and dust). Broadly, BT is categorized as either orthodox or CTC. Oolong tea, which is manufactured mainly in Taiwan, is produced with a shorter fermentation period than black tea and is said to have a taste and colour somewhere between GT and BT [38–41]. Tea is generally exported with minimal processing to importing consumer countries, where it is blended and packaged by the tea companies [42]. Generally, unblended black teas are named after the region in which they are produced, such as Ceylon or Assam tea, while blended teas include a variety of teas, often from countries of different origin. The two tea products most widely consumed are GT which is primarily consumed in Southeast Asia and BT which is the main tea beverage in India, Europe, Russia, North America, the Middle East, Indonesia, North Africa, Chile, Hong Kong, Australia, and New Zealand. BT represents over 90% of all tea sold in the West [1]. The consumption of oolong tea is mostly restricted to China and Taiwan [43].

There are two main varieties of* Camellia sinensis* that make up most of the tea consumed internationally,* Camellia sinensis* var.* assamica*, or the broad-leaf variety of the tea plant, and* Camellia sinensis* var.* sinensis*, or the small-leaf variety of the tea plant [44, 45]. GT is most often sourced from the small-leaf variety because it has a sweeter taste than the broad-leaf variety that is usually used for BT production. Tea plants of the broad-leaf variety are quickly growing plants or either tree or bush form, suitable for warm, tropical, and subtropical environments and are predominately grown in Southwest China, Northern Laos, Northern Vietnam, Myanmar, Cambodia, North Eastern India, and Africa. The small-leaf variety is more suitable for colder, temperate climates and is predominately grown in eastern China, Taiwan, Japan, India's Darjeeling, and tea growing countries outside of Asia [46, 47].

Although tea is the most popular beverage consumed by two-thirds of the world's population [48], the consumption of tea remains much less in most of the Americas, tropical Africa, and Nordic, Baltic, and Southern European countries [1, 49]. While consumption of tea in the USA is among the lowest in the world [1], the USA is unique for its large consumption of iced tea, prepared at home or sold as canned or bottled beverages. According to the Tea Association of the USA the canned/bottled tea segment comprised just under 50% of the market share in 2016, with bagged/loose leaf tea comprising approximately 23% market share [50]. Iced tea is prepared by cooling traditionally brewed BT, but it is sometimes prepared by the prolonged steeping of tea at room temperature or in chilled water. Cold water-soluble instant teas are also used [38]. Instant tea is a powder that water is added to, in order to be reconstituted into a cup of tea, and is produced from the fermented juice of BT leaves through hot water extraction, aroma recovery, soluble solids concentration, aroma restoration, and drying to obtain concentrate tea granules [51].

BT consumption is the staple of the Australian and NZ diet since both countries were colonized by British settlers. Notably, by the mid-nineteenth century, colonial Australians were using between four and five kilograms of tea per capita annually, the highest consumption of tea per capita globally [52]. By the beginning of the twentieth century, NZ had the second highest annual per capita consumption of tea in the world (3.0 kg) next to Australia (3.1 kg) [49]. Indeed annual per capita tea consumption was 75-fold higher than India (0.04 kg), 15-fold higher than Japan (0.2 kg), 10-fold higher than France, and 7.5-fold higher than the USA (0.4 kg) [49].

Paradoxically, during the 1960s when water fluoridation was actively expanding in NZ, annual per capita tea consumption of tea was even higher at 3.35 kg per person per year [53]. Despite the recent decline in per capita consumption of tea, BT remains one of the most frequently consumed beverages in NZ consumed by 80 percent of adult New Zealanders aged 22 to 55 years [54]. In 2012, per capita consumption of BT per person per year was reported to be 8-fold higher in NZ compared to the USA [1]. Per capita consumption in Australia was 5-6-fold higher than the USA [1]. The differences in per capita consumption are reflected in population based case control studies conducted in Australia and the USA. For example, Nagle et al. (2010) reported that 60% of Australian women aged 18–70+ years consume 1 to 4 or more cups per day [55]. In comparison, Song et al. (2008) reported that the frequency of women aged 35–74 years in the USA who drank one or more cups of BT per day was 10 per cent [56]. Similarly, the 2008 Food Standards Australia NZ Total Diet Survey (TDS) identified that for Australian adults aged 30–69 years mean daily consumption of tea was approximately 1 L [33]. At the same time the USA National Health and Nutrition Examination Survey (2007/2008) reported that the mean daily consumption of tea among adult non-Hispanic whites age 20 years and over was 236 ml per day [57].

In their review of F in drinking water released in March 2006, the National Research Council (NRC) of the USA National Academies of Science (NAS) recommended that the USA Environmental Protection Agency (EPA) updates its F risk assessment to include new data on health risks and better estimates of total exposure [2, 58]. The NRC identified subpopulations that might be particularly susceptible to the effects of F including people with high activity levels (e.g., athletes, workers with physically demanding duties, and military personnel); pregnant or lactating women; individuals with renal disease, because of their high fluid intakes and compromised ability to excrete F which can result in greater accumulation of F in their bodies; the elderly, because of their long-term accumulation of F into their bones; and individuals with medical conditions that can make people more susceptible to the effects of F, such as individuals with thyroid disorders or individuals with compromised immune systems [2].

The NRC recommended a wide range of additional epidemiology, toxicology, clinical medicine, environmental exposure, and biomonitoring assessments that need to be undertaken in order to fill data gaps. Further studies were recommended in identifying sources of F and determining bone, soft tissue, plasma, and urinary F levels and further studies to clarify the relationship between F intake, fluoride concentration in bone, clinical symptoms of skeletal fluorosis, and other factors that may influence the risk of bone fractures [2]. The NRC also recommended that carefully conducted studies of exposure to F and emerging health parameters of interest (e.g., endocrine effects and brain function) should be performed in populations exposed to various concentrations of F. Further studies were recommended to investigate the effects of F on brain, kidney, liver, and immune system function as well as in vivo human genotoxicity studies in US populations or other populations with nutritional and sociodemographic variables similar to those in the United States. Additional studies were also recommended to investigate the association between F intake and thyroid disease, calcium metabolism, pineal function, and development of glucose intolerance and diabetes [2].

In the USA, fluoridation of community drinking water for prevention of dental caries commenced in 1945, reaching 3 per cent of the population by 1951 and 74.6 per cent in 2012 [59, 60]. When water fluoridation commenced in the USA, drinking water was considered the main dietary source of F intake for the population. Today, community water fluoridation and F toothpaste are considered the most common sources of F exposure in the USA [61]. In 2010, an exposure assessment conducted by the EPA concluded that the use of fluoridated water for commercial beverage production and the increase of F in solid foods because of fluoridated commercial process have likely resulted in increased dietary F intake adding to the risk for overexposure [62]. In dietary exposure assessments, uncertainty results from limitations in scientific knowledge, including the factors that determine exposure, and data availability [63]. Risk assessments can address such questions as to whether a risk to health will be reduced if certain actions are taken and, if so, by what magnitude and whether new risks might be introduced when such actions are taken [64]. However, risks cannot be reliably estimated if exposures and their uncertainties are not properly characterized and sufficiently quantified [64]. Recently, due to potential concerns associated with cumulative F exposure and to ensure a high level of consumer protection, the USA Public Health Service (PHS) finalized a recommendation for lowering the levels of water fluoridation programs to 0.7 mg/L for the whole USA [65, 66]. The earlier PHS recommendation for F concentrations was based on outdoor air temperature of geographic areas and ranged within 0.7–1.2 mg/L. However, fewer than 1% of fluoridated systems in the USA used a F concentration of 0.7 mg/L. Implementation of the new recommendation is expected to lead to a reduction of approximately 25% in F intake from drinking water alone and a reduction of approximately 14% (range 5%–29%) in total F intake [66].

In NZ, the USA PHS endorsement of water fluoridation in 1951 generated consequences, laying the foundation for implementation of water fluoridation in the first community outside of North America, when Hasting Borough Council implemented fluoridation in 1953 [67]. However, in contrast to the USA, Harrison (1949) identified that BT consumption was the major dietary source of F among New Zealanders [68]. Subsequently, in 1957, the report of the NZ Commission of Inquiry appointed to inquire into and report upon fluoridation endorsed the fluoridation of municipal water supplies [69]. However, the contribution of tea to dietary F intake was not considered. Following publication of this report, fluoridation rapidly expanded in the 1960s extending to Lower Hutt, Palmerston North (1962), Wellington (1965), Auckland (1966), Hamilton (1966), Dunedin (1967), and New Plymouth (1971) [70]. The Drinking-Water Standards for NZ (2008) include a maximum acceptable value (MAV) for F of 1.5 mg/L and recommended a target F range of 0.7–1.0 mg/L for oral health reasons [71]. Actual F concentrations in municipal water in NZ have been found to average about 0.8–0.9 mg/L in fluoridated areas and around 0.15 mg/L in nonfluoridated areas [72]. At present, there are some 84 water supplies in the NZ where F is intentionally added [69], providing fluoridated drinking water to a little over half of the NZ population [73]. Colquhoun (1987) examined child dental health differences based on fluoridation status and demographic factors in NZ and found that when similar populations were compared, there was no obvious dental health benefits related to fluoridation [74]. More recently, the 2015 NZ Health Department statistics on 46,948 five- and 46,942 eight-year-old children revealed a 0.89% caries-free difference at 5 and 1.27% at 8 years, respectively, between fluoridated and nonfluoridated communities [75].

In 2015, the NZ Government started the legislative process to remove decision-making on fluoridation away from local authorities which will make fluoridation a legal requirement for local authorities if directed by the district health boards [76]. If these legislative changes are implemented, the percentage of the NZ population provided with fluoridated water will significantly increase. This will result in increases in cumulative F intake and may increase the risk of chronic fluoride exposure, particularly among tea drinkers. The NRC (2006) reported that, due to the substantial amounts of F in commercial tea brands, a combination of exposures can lead to higher than expected F intake, which may contribute to musculoskeletal problems [2]. As the borderline between toxic and beneficial intake as regards the prevention of dental caries is very narrow, the average daily F intake of a community must be investigated well in order to estimate whether or not there is reason to add F to the drinking water and, if so, what is the safe but adequate concentration [77].

Cressey (2010) found that NZ infants fully formula-fed on formulae prepared with optimally fluoridated water (0.7–1.0 mg/L) exceeded the upper level of intake (UL) for F and are at increased risk of dental fluorosis [78]. Previously, Colquhoun (1984) reported a highly significant difference between the incidence of dental fluorosis among children living in fluoridated and nonfluoridated areas of NZ [79]. The prevalence of dental fluorosis in fluoridated communities was 24.9%, ranging from 22.7 to 27.7% depending on socioeconomic factors, with the highest prevalence found among children from lower socioeconomic areas. In comparison, the prevalence of dental fluorosis in nonfluoridated communities where the drinking water source was groundwater was 4.9% and 2.9% in nonfluoridated communities where drinking water source was rainwater. Notably, among the children examined in the nonfluoridated area, the most advanced case of dental fluorosis was a 10-year-old Maori male, due to consumption of tea from an early age [79]. Mackay and Thomson in a 2005 study of 436, 9-10-year-old, children in Southland, New Zealand, found that among children continuously residing in a fluoridated area up to the age of 4 years 32.1% had diffuse opacities, consistent with dental fluorosis, compared to 19.0% for children with no or noncontinuous residence in a fluoridated area [80]. More recently, the 2009 Oral Health Survey reported that, using Dean's Index of Fluorosis, 44.5% of 8–30-year-olds had some degree of dental fluorosis [81]. This data illustrates that the prevalence of dental fluorosis in NZ has increased significantly since the 1980s. These findings raise other implications as chronic exposure to F in infancy will result in accumulation of F in skeletal tissue. Thus, as people age, their bone F levels will be higher than for similar aged individuals residing in nonfluoridated communities.

In addition to tea and fluoridated water, other sources of F exposure include other beverages produced from fluoridated water (beers, coffee, soft drinks, and fruit juices); pesticide residues in foods, foods processed or cooked in fluoridated water; foods grown in soil containing F or irrigated with fluoridated water; consumption of foods with elevated F levels (i.e., seafood and processed chicken); foods cooked in Teflon cookware; tobacco consumption; use of fluoridated toothpaste; fluoridated mouthwash; use of medical inhalers containing fluoridated gases, and fluoridated medications, in addition to other environmental or occupational exposures to F.

Typical fluoridated medications that have been found to contribute to fluoride burden include fluoroquinolone antibiotics [82]; the antifungal medication voriconazole [83]; the antimetabolite drug fluorouracil [84]; the antiarrhythmic drug flecainide [85]; anti-inflammatory drugs diclofenac, niflumic acid, flufenamic, and antrafenine [86, 87]; the antimalarial drug 5-fluoro-amodiaquine [88]; antipsychotic medications such as haloperidol [89]. There is evidence to suggest that Teflon products may significantly alter the F uptake in prepared foods [90]. There is additional evidence that consumption of beer and wine can contribute significantly to F intake, particularly among habitual users [91, 92]. Evidence suggests that smokers will be exposed to higher chronic exposure to fluoride than nonsmokers [93–97]. Recently, Gondal et al. (2015) reported that the fluoride content in four different brands of cigarettes commercially available in Saudi Arabia ranged from 233.9 to 360.2 ppm [98]. However, due to limited awareness on the fluoride level in tobacco, it is usually ignored when calculating the total dietary intake of fluoride [98].

Regarding food production, it is noteworthy that NZ agricultural soils are already exceeding the thresholds protective against chronic fluorosis in grazing animals, due to large-scale use of phosphatic fertilizers rendering large areas of agricultural lands unsuitable for particular types of primary production [99]. Accumulation of F in soils has implications for not only grazing animals but also soil microbial activity and fertility [99–102], soil acidification [103], and the potential for increased dietary intake from cereals and vegetables [104, 105].

The relationship between F accumulation in agricultural soils from large-scale application of phosphate fertilizers is a relatively new phenomenon [99] and has many parallels to water fluoridation. Both progressed in the second half of the 20th Century and the consequences of long-term accumulation have largely gone undocumented. Indeed, water fluoridation provided an opportunity for the fertilizer industry to dispose of otherwise hazardous industrial waste products by reusing these materials for the purposes of water fluoridation. In doing so waste became a valuable commodity. Because F does not biodegrade, the contribution from fertilizer inputs, industrial and environmental emissions accumulate in the environment. Treated wastewater and sewage sludge from water treatment plants treating fluoridated wastewaters also contain F, which ultimately adds to the environmental burden on ecosystems. The use of fertilizers and fluoridated chemicals in tea production has also increased in the last half century to meet the challenges of increased yield and the management of tea pests associated with monoculture production. This has led to concerns regarding pesticide residues in tea products and its toxic hazards to consumers [106]. Regulatory agencies have recently fixed the maximum residue levels and/or permissible limits of pesticides/chemicals in teas [107], and chemical characteristics have been defined by the International Organization for Standardization (ISO) for BT and GT [108, 109]. However, there is no requirement currently to measure the F level in tea, and no maximum residue levels (MRLs) have been established for F levels in tea products.

Thus, in order to make safety evaluations, it is necessary to determine the F content of various products of black tea available to consumers [5]. A recent review of the health effects of water fluoridation in NZ reported that individual exposure due to the consumption of tea can range from 0.04 to 2.7 mg/day [110]. Unfortunately, the information base on which to estimate exposures and doses associated with tea consumption in NZ is completely lacking. Moreover, the suggested upper limit is significantly lower than that reported in scientific literature internationally [5–10].

Therefore, the aim of our study is to determine the F levels in the most widely available and consumed tea beverages in NZ, thereby improving the status of scientific knowledge and the treatment of uncertainty in dietary risk assessments for F intake. The study further aims to access the range of likely human exposures to F from BT consumption in NZ, while also reviewing the factors that influence the F content in tea products and infusions. In addition, the study will look at temporal changes in F exposure from tea and address bioavailability of F from tea. In this paper, we provide an overview of the international literature on the relationship between F intake, chronic pain, musculoskeletal disorders, and arthritic diseases and discuss its relevance to NZ.

## 2. Materials and Methods

All 18 tea products employed in the study were procured as boxes of teabags from supermarkets, retail outlets, and health food stores between July and August 2016. The samples represent the clear majority of BT products purchased or available to NZ consumers. We did not consider other types, such as ready to drink bottled or canned iced tea, green, oolong, white, yellow, or herbal tea, which are not widely consumed in NZ. The characteristics of the analysed teas are listed in Table 1. The samples were prepared as per our previous study for measuring F content in black tea products in the Republic of Ireland [10]. Briefly, prior to analysis duplicate teabags were randomly selected from each packaged tea product, weighted, and placed in a porcelain cup. Municipal fluoridated tap water was boiled in an electric kettle, and approximately 200 mL of water was poured into the cup and a single teabag allowed too steep unagitated for a period of 5 minutes (according to the tea industry's recommended brew time). It is observed that the temperature decreased from 100 to 70°C during the first five minutes. After five minutes, the teabag was extracted and the infusion was allowed to cool to room temperature. The scenario was repeated in for each brand of tea tested. Controls were prepared by using boiled deionized water, such that 54 samples were collected in total. The F determinations were conducted potentiometrically after addition of total ionic strength adjustment buffer (TISAB) using a F ion-selective electrode (Extech FL700) [10]. The Extech FL700 has a reported resolution of 0.1 mg/L and overall accuracy of ±3%. The TISAB buffer tablet was supplied by Extech (FL704 TISAB tablets) and added to a 20 ml aliquot of tea infusion placed in a polyethylene plastic sample container prior to measurement. A series of F standards was prepared by using a 100 mg/L F Eutech standard solution (Eutech Instruments, F Standard Solution Code number ECSCSFL3BT) by diluting appropriate volumes to 100 ml with deionized water. Then, the electrode was calibrated to concentrations of 0.0, 0.5, 1.0, 5.0, and 10.0 mg/L. Prior to sampling and at the end of each group of five samples, the electrode was rechecked for accuracy.

The results of these analyses are presented in Table 1. For the duplicate tea infusion samples, the mean value is provided. The variability between duplicate samples was very small, generally within 0.1 mg/L and is therefore not reported.

## 3. Results

For the purposes of examining F intake from tea infusions, it is the F content in infusions prepared with tap water that is of interest. When prepared with tap water, the F concentration in tea infusions ranged from 0.9 to 3.9 mg/L with a mean of 1.9 mg/L. Significant differences in F concentrations were observed between tea brands, with NZ economy and NZ own brand supermarket blended tea products having the highest F concentrations. Variations were also observed in the weight of teabags, which were found to range from 1.7 g to 3.3 g. Notably, the highest F concentration in tea infusions (3.9 mg/L) was found in the product with the maximum weight of tea (Bell Kenyan Bold). Notwithstanding this, the second highest concentration (3.4 mg/L) was also found in Bell Original Tea where the weight of tea was comparable to other tea products tested.

It is important to note that Bell teas have the largest market share in NZ [111], representing the main brand of choice among consumers [54]. Consequently, these products would be most representative of consumer exposure. Notably, the F content in tea infusion made from these products is 3-4 times higher than the accepted level in drinking water in NZ. Variations were also observed in the F concentrations of tea infusions based on country of origin with the highest levels found in teas originating from Kenya and the lowest in teas originating from Sri Lanka. However, none of the products provided information on the F content.

Overall, the present study found that the F ion concentration in tea infusions prepared from 18 different black teabag commodities exceeded the new USA DHHS recommendation for a single level of 0.7 mg of F per litre of water [66]. In addition, sixteen products (89 per cent) exceeded the upper range permitted for fluoridated drinking water in NZ (1.0 mg/L) and nine products (50%) exceeded the World Health Organization (WHO) maximum permissible limit for F in drinking water (1.5 mg/L).

While there are no internationally safety standards for the F content in tea beverages, 55% of the tea infusions exceeded the permissible level in the US for imported bottled water (1.4 mg/L) [112] and 50% exceeded the maximum permitted level requiring labelling and safety precautions for bottled mineral water under European regulations (1.5 mg/L) [113].

In NZ, Foods Standards Australia New Zealand has established a maximum level (ML) of 1.0 mg/L (naturally occurring and added) for packaged potable water [114]. Based on this recommendation, 90% of the teas tested would exceed this ML when prepared with tap water containing 0.7 mg/L. Moreover, all of the tea products would exceed this ML when tap water contains more than 0.7 mg/L. As expected, the use of fluoridated tap water at 0.7 mg/L was found to significantly increase the measured F content in all tea infusions. In NZ, the Ministry of Health recommends the adjustment of F to between 0.7 and 1.0 mg/L in drinking water [71]; thus, the value of 0.7 mg/L in this study represents the lower range found in fluoridated drinking water in NZ.

Notwithstanding the contribution of fluoridated water, when prepared with deionized water, the F concentration was less than 0.5 mg/L in 2 out of 18 samples of the tea infusions, 0.5–1.0 mg/L in 5 out of 18 tea infusions, and higher than 1.0 mg/L in 5 out of 18 tea infusions.

### 3.1. Comparison with Previous New Zealand Studies

The only other study to publish F levels in BT in NZ dates to 1949 [68]. In this study, the author reported that almost all teas imported to NZ at this time were from either Ceylon (Sri Lanka) or India. Samples of both types of orthodox loose tea (4 in total) were obtained and the F content was measured in infusions. The weights of tea per infusion were selected to cover the range for most tea tastes and varieties. The weights of tea to water were 6.75 g/L, 10 g/L, and 15.0 g/L to represent light, medium, and strong tea. Samples were infused for 3 and 10 minutes with tap water with a F content of 0.05 mg/L. At 3 minutes' infusion time, the F content was reported to range from 0.46 to 1.26 mg/L increasing with strength of brew. At 10 minutes, the F content was reported to range from 0.48 to 1.69 mg/L. The lowest F levels were found in North Indian teas and the highest in Ceylon teas. The author reported that, based on the daily ration of 8.1 g of tea per day, F intake from tea would range from 0.5 to 1.0 mg/day [68].

### 3.2. Exposure Assessment

As we previously reported [10], there is some controversy regarding recommendations on Adequate Intake (AI) for F since there are no signs of F deficiency which have been identified in humans, and F has no known essential function in human growth and development [115]. Evidence over the last 20 years has demonstrated that the cariostatic effect of F is topical on the tooth surface and ingestion is not required [116–118]. In 2011, the EFSA commissioned the University of East Anglia to examine the scientific data from which dietary reference values (DRVs) for F may be derived. The comprehensive review found that there were relatively few studies of good quality regarding F intake, accumulation, and/or health endpoints. The review concluded that there was a lack of high quality evidence upon which DRVs may potentially be based for F [119]. Notwithstanding this, the EFSA have recommended that the AI for a healthy adult woman is 2.9 mg/day and 3.4 mg per day for a healthy adult male [120]. In the USA, the Institute of Medicine has recommended that the AI is 3.0 mg and 4.0 mg per day, respectively [121]. In the EU, the Tolerable Upper Intake Level (UL) for F has been established at 7 mg/day for adults [122].

According to the EFSA (2010), an AI is the average observed daily level of intake by a population group (or groups) of apparently healthy people that is assumed to be adequate, while chronic intakes above the UL may be associated with an increased risk of adverse effects [123]. In general, the AI for F are based on estimated intakes that have been shown to reduce the occurrence of dental caries maximally in a population without causing unwanted side effects including moderate dental fluorosis [122]. The AI and UL apply to intake from all sources including water, beverages, foodstuffs, dental health products, and drugs. The Scientific Committee on Health and Environmental Risks (SCHER) reported that the emerging picture from all risk assessments conducted on F is that there exists a narrow margin between the recommended intakes for the prevention of dental caries and the upper limits of exposure [124]. In view of the different health, nutritional status and habits of individuals in addition to the differing consumption of drinking water and the uncontrolled intake of F from other sources, such as tea, medications, or occupational exposures, it is difficult to accurately define an AI or UL for a population without excluding sensitive subgroups of the population. To illustrate this dilemma, the intake of F for individuals with low iodine status or poor renal function poses significantly higher risk of negative outcomes than for healthy individuals [2].

While this study is the first to measure F concentration in a wide range of commercial BT products available in NZ, it is important to note that the exposure assessment presented here is specific to adult consumption of tea in NZ and excludes all other sources of dietary F intake. Clearly, any estimation of F intake from tea is dependent on total volume consumed. Recent consumer surveys in both Australia and NZ have found that the clear majority of tea drinkers prefer to drink tea in a mug rather than a cup [125, 126]. A standard cup size in Australia is the same as the USA (250 ml); however, the market trend in the past two decades is for larger mug sizes ranging from 350 to 600 ml.

In this study, we have selected 350 ml to be representative of mug volumes. The variation in cup sizes is important in characterizing risk. For example, the standard tea cup size in Turkey is just 75 ml [127], which is significantly less than that for Western countries. Notably, per capita consumption of black tea in Turkey is reported to be approximately twice that of NZ and three times that of Australia [1]. However, black tea intake, in terms of daily volume of tea infusion consumed, is significantly higher in Australia [33] than Turkey [127]. This reflects the differences in cup sizes as well as the differences in tea products used to prepare tea infusions. For example, in Turkey, orthodox loose tea remains the tea product of choice [127].

In this current study, the dose of F in tea infusions for each brand of tea was calculated for a range of consumption scenarios ranging from 1 cup to 14 cups per day based on data on tea consumption among pregnant women in NZ reported by Ford et al. (1998) [128].

The results are provided in Table 2. It is notable that, depending on cup size, the consumption of 14 cups of tea per day would equate to 2.8 to 3.5 L of tea per day. Based on this NZ data, we have assumed that 3.5 L per day is the reasonable maximum exposure (RME) in our study for tea consumption in NZ. The RME is considered conservative as several studies in the United States have reported individuals consuming 3.5–7.5 L of BT per day [129–131].

In Table 3, the estimated F intake per mug of tea consumed is presented. The F levels reported represent the measured F concentrations in tea infusion prepared with drinking water containing 0.7 mg/L F.

Considering that the upper level of F in drinking water permitted in NZ is 1.0 mg/L, an approximate estimate of F intake has also been provided to allow for the differences in F levels. Thus, the reported levels represent the lower and upper range for the 18 products tested.

Based on our results, assuming the consumption of one normal mug of tea liquor/infusion per day, we estimate that the daily F intake will range from 0.1 to 1.1 mg/day for nonfluoridated communities and 0.3 to 1.5 mg/day for fluoridated communities. Assuming that the consumption is of 2–4 mugs, we calculate the daily intake from tea to be in the range from 0.3 to 4.5 mg/day in nonfluoridated communities and 0.6–5.9 mg/day in fluoridated communities. For high tea drinkers consuming of 6–10 mugs per day, the F intake would range from 0.8 to 11.2 mg per day for nonfluoridated communities and 1.9 to 14.7 mg/day for fluoridated communities.

Taking the highest tea intake reported by Ford et al. (1998) among NZ pregnant women (14 cups per day) [128], F intake from tea would range from 1.4 to 11.2 mg/day in nonfluoridated communities and from 3.2 to 14.7 mg/day in fluoridated communities.

However, exposure assessment should be representative of actual consumer preference. As Bell tea has the largest market share in NZ [111], representing the main brand of choice among consumers [54], the intakes associated with the two Bell tea products more accurately reflect consumer preference and exposure. Taking this into consideration, we have evaluated the contribution of the two Bell tea products to AI and UL intake reference values. The percentage contribution of each brand to AI and UL was calculated using Microsoft Excel. The values reported reflect the F content of drinking water (0.7–1.0 mg/L) as recommended by the NZ Ministry of Health.

A summary of exposure and risk estimates for F intake is presented in Table 4. The values do not include additional dietary exposure from other beverages, food, toothpaste or dental products, medications, or occupational exposures. The results reported in this study demonstrate that a single mug of Bell Kenyan tea when prepared with fluoridated tap water can provide half the daily AI for a healthy female, excluding all other sources of F intake. Two mugs of Bell Kenyan tea can provide the full AI. The consumption of 10 mugs or 14 cups of Bell Kenyan tea can exceed the AI 5-fold and the UL 2-fold for a healthy adult female.

For Bell original black tea, the results are similar though marginally lower. For an adult male, one mug of Bell Kenyan tea per day can provide up to 44% of the recommended AI with Bell Original Tea providing up to 38% of the AI. Two mugs per day would provide between 79 and 85% of the AI and up to 37% of the UL for a healthy adult male. Notably, for the two major supermarket own brand BT bags, the results would be similar, though marginally lower.

## 4. Discussion

### 4.1. Factors That Influence the Fluoride Content in Manufactured Tea and Tea Infusions

Many factors influence the concentration of elements including F in tea plants and its product including cultivar type and growth conditions (soil pH; the F and aluminium contents and their forms in soils; rainfall, altitude, air, and soil pollution from industrial sources and urban activities), horticultural practices (the application of chemical and organic fertilizers, use of pesticides and soil conditioners, the F content of water used for irrigation, mechanical or hand plucking, and age of leaves), the period of withering, and the mechanical processes used in the treatment and packaging of tea [36, 132–150].

The primary factors affecting the amount of total water-soluble F in tea infusions are the temperature of water used for brewing, particle size, brewing time, water hardness, and background levels of F in drinking water [138, 143, 151–156].

Typically, the higher the temperature of water, the higher the diffusion of F; the smaller the particle size the greater the rates of diffusion. In this context, it is worth noting that one of the most widely reported incidences of chronic tea ingestion and skeletal fluorosis from tea involves that of an American woman as reported by Kakumanu and Rao (2013). In this case study, it was reported that the patient used 100 to 150 teabags daily to prepare a tea infusion for consumption [157]. However, the authors did not clarify how the tea was brewed and no measurement was made of the F content in the tea infusion. It was subsequently indicated (D. S. Rao, personal communication, December 23, 2014) that the patient made iced tea by steeping the teabags in cold water at room temperature.

To better understand how water temperature affects the diffusion of F from teabags, we compared the F levels in different BT infusions from commercial teas with hot (boiling water for 5 minutes) or cold (room temperature for 20 minutes) water. The infusible F level in the cold brew using 50 teabags (each teabag containing 3.5 g of tea) in 3 L of tap water after 20 minutes was 3.2 mg/L, compared to 5.6 mg/L for a single teabag of the same brand left to brew in boiling water for 5 minutes (data not provided). The standard weight of a teabag in the USA is 2 g; thus, the 50 teabags used in this experiment would be equal in weight to 75 teabags in the USA. What this demonstrates is that it is not the quantity of teabags that is relevant but the F content in the tea infusions, which is primarily influenced by the temperature of brewing.

It is also important to be aware that, in the Far East (particularly China and Japan), the brewing temperature of green tea is typically 70~80°C. In contrast, black tea is brewed using boiling water. In addition, the Chinese believe that the second infusion tastes better than the first. This has led some green tea manufacturers to recommend that the first infusion be discarded and the second infusion consumed [140]. According to infusion experiments, the F content in the second infusion is approximately 20% of that present in the original infusion [153]. Hence, the F content in the original tea leaf or original infusion may not be representative of actual F intake. Clearly cultural influences in Asia in how tea is prepared and consumed vary significantly from Western countries, and the culture of preparing tea by continuous or repeated infusions prior to consumption can greatly influence F exposure.

Regarding Western practices of preparing tea in a pot or mug significant differences may occur depending on individual consumer habits and tastes. In experimental settings, the majority of studies use a steep time of 5 minutes to represent the realistic maximum time used by consumers and recommendations by tea brands. However, in a real-life scenario, a general consumer is unlikely to monitor the exact amount of time that their tea is steeping. Steep times will likely increase when preparing a pot of tea, compared to use of instant teabags in a mug, and use of more than one teabag is a widespread practice when preparing a pot of tea. Individual variations in tea making in Western countries among teabag users can also differ significantly. Some add boiling water and just leave the teabag(s) to float for a given time. Others move the teabag up and down (dunking) or agitate the teabag with a spoon. Ducking or agitating the teabag will increase the diffusion rate. Hence, higher F levels in infusions are expected when preparing tea in this manner. Past studies have shown that most F is rapidly extracted from teabags in the first minute and slowly increases with time. The concentration of total F extracted at 5 minutes is comparable to that at 10 minutes and 15 minutes [151, 152].

Previous studies have shown that the addition of milk to tea (English style tea) has no effect on the leachability of F from tea [158–161]. Clearly, as identified in our study, the use of fluoridated tap water to make tea infusions contributes significantly to the total F content in tea beverages. Another factor that influences F content in infusion is cup size. The larger the cup size, the greater the volume of beverage consumed and the greater the potential F intake. A cup of tea could be 30 ml as in Senegal [162] and China [163]; 80 ml as in Saudi Arabia [164]; 80–100 ml as in India [165]; 100 ml as in Japan [166], Turkey [167], and Iran [168]; 135 ml as in Italy [169]; 240 ml (8 oz mug) as in the USA or in the case of iced tea the amount is larger (360 ml) [170]. In the RoI, UK, Australia, and NZ, tea is mainly consumed in mugs. There are no standardized mug sizes; however, they generally range from 250 to 400 ml. It is noteworthy that studies on the extraction efficiencies of caffeine from brewed teas found that when BT or GT teabags were brewed in a larger serving size, the extraction efficiencies increase [171].

### 4.2. Temporal Changes in Exposure to Fluoride in Tea Beverages

In exposure science, one must consider temporal changes. In recent decades, there have been major changes in production practices in tea cultivation and harvesting that contribute to higher F levels in tea products. The use of agrichemicals and mechanization of harvesting has increased dramatically in recent decades and more recently climate change has also influenced tea production and quality. However, in considering temporal changes, one must also examine if the sources of tea have changed in recent decades. In this context, it is important to note that, since the 1960s, the source of BT marketed and consumed internationally has dramatically changed. Prior to the 1960s, the most BT available in Western countries originated in India and Sri Lanka. To meet demand for cheaper quality tea and increased production, tea cultivation increased dramatically in East Africa (principally Kenya) from the 1960s and international tea companies increasingly relied on Kenya to source BT products. Tea production has also increased significantly in Argentina to meet the demands of USA market [172]. Several studies have found a clear distinction in the heavy metal characteristics of teas produced in African (Kenyan) tea compared to Asian teas (China, Japan, Sri Lanka, and India) [137, 139, 173]. This could also have implications for residue levels of F in tea.

It is noteworthy that food safety authorities in China recently imposed restrictions on most of Kenya's tea products due to potentially hazardous concentrations of F found in some of their tea products [174]; however, to our knowledge, no restrictions have been implemented by any other country. In our previous study, we also reported that the highest concentrations of F in BT products available in Ireland were found in Kenyan teas, which represent the most blended BT products in Ireland and the UK and the lowest in teas from Sri Lanka [10]. In this study, we also found that NZ teas originating from Kenya had the highest F level and teas originating from Sri Lanka had the lowest F content. While tea from Kenya has been the major source of tea in Ireland since the 1970s, it has not been reported in literature when tea sourced from Kenya was first introduced to the NZ tea market.

A second factor that has increased consumer exposure to F from BT was been the rapid rise in popularity of teabags, from the 1970 onwards. The CTC process has become especially popular as the teabag market developed [175]. Prior to 1970, orthodox leaf tea was the dominant form in which tea was consumed. Today, consumption in industrial countries is now predominantly in teabag form. For example, in the Republic of Ireland, 95% of the tea is sold in teabags (CTC black tea) with the orthodox loose tea leaf market representing just 3 to 4% [176]. NZ is similar to the UK and Ireland, with 94% of consumers purchasing black teabags [54]. In contrast, orthodox loose tea is still the predominant type of tea consumed in many Asian and Middle Eastern countries. For example, in Turkey, teabags constitute just 5–10% of the tea market [177].

Considering that higher F content has been found in BT infused in teabags than loose orthodox BT [6, 134, 150], it would appear that the introduction of the black CTC teabags to international tea markets post-1970 and the change in origin of imported tea significantly altered population exposure to F. This is evident in the differences in F levels reported in our study with those reported for BT products in NZ during the 1940s [68]. In NZ, population exposure was further exacerbated by the rapid expansion of water fluoridation in the 1960s.

### 4.3. Bioavailability of Fluoride from Tea

The first study to accurately quantify bioavailability of F by tea was by Toyota (1979) [178]. In this study, the authors demonstrated among subjects aged 15 to 59 years of age that the consumption of 300 ml of orthodox BT infusion containing 1.40 mg of F was found to increase background serum F levels 5-fold (0.526 to 2.78 *μ*mol/L) within 1 hr. Within 3 hrs, urinary F concentrations increased 7-fold from 0.5 mg/L to 3.5 mg/L and declined to 1.5 mg/L six hours after consumption [178]. More recently, Chan (2014) undertook a bioavailability assessment of F in three UK black tea infusions prepared from two different brands of teabags and one brand of loose leaf tea. The order of percentage F bioaccessibility in the tea products and infusions was economy BT bags > BT bags > pure orthodox BT leaf tea. For the economy BT bag products, up to 103.8% of the F appeared to be available for absorption in the gastric fraction and 109.4% in the gastrointestinal fraction. In the leaf tea products, the results were 83.2% and 97.5%, respectively [179]. These findings somewhat confirm previous results from animal studies which demonstrated that F uptake in bone was significantly higher from tea infusions prepared from teabags with a F content of 2.6 mg/L than from fluoridated tap water containing the same F content after a period of just 30 days [180]. Another factor that contributes to F bioavailability is caffeine. Chan et al. (1988) found that coexposure to caffeine and F resulted in significantly increased bioavailability of F in humans, as measured by plasma F levels, compared to subjects exposed to F alone [181]. It is important to note that tea is also a rich source of caffeine [182].

Based on this evidence and that previously discussed we hypothesise that the bioavailability of F from BT manufactured by CTC is higher than orthodox tea. We further suggest that this enhanced bioavailability may also be associated with the minute tea leaf particle size in CTC tea which can be released from the teabag as dust into the tea infusion and ingested, thereby contributing to the overall exposure of a consumer.

### 4.4. The Association between Fluoride Intake, Tea, and Skeletal Fluorosis

Once F is absorbed by ingestion, it passes into the blood for distribution throughout the body and for partial excretion [183]. However, inorganic F is a persistent bioaccumulator, and the ever-increasing use (and release) of F compounds in the environment should be of long-term concern in population subgroups who are most susceptible and, therefore, most “at risk” [184]. The WHO (2000) reported that skeletal fluorosis is associated with a systemic uptake exceeding 5 mg/day in a relatively sensitive section of the general population [185]. Thus, the question of the bioavailability and additive toxicity of F from tea is important particularly in countries with water fluoridation.

The USA NRC (2006) reported that, in the absence of other sources of F (i.e., tea or toothpaste), the consumption of drinking water with a F level of 1 ppm, bone F levels can reach 2,500 mg/kg in twelve years and to 3,000 to 4,000 mg/kg over longer periods [2]. Moreover, the committee observed that evidence suggests that preclinical bone changes and symptoms of clinical stages I and II skeletal fluorosis may occur with bone concentrations above 3,500 mg/kg [2]. Stage II skeletal fluorosis is associated with chronic joint pain, arthritic symptoms, slight calcification of ligaments, and osteosclerosis of cancellous bones [2]. In studies, which have measured bone F levels in nonfluoridated and fluoridated communities in the USA, UK, Canada, and Finland, significant variations were observed. For example, Weatherell (1966) measured the F levels in femoral compacta from humans of different ages who lived in communities supplied with drinking water containing < 0.5 ppm F in various locations in England and Rochester, New York. Significant differences were found between the two populations, with F accumulating to 4,000 ppm among the English subjects compared to less than 1,000 ppm among residents in Rochester [186]. Since drinking water in both communities was nonfluoridated (<0.5 ppm), the fourfold difference in bone F levels reported clearly reflects the higher consumption of tea in England compared to the US [1]. As noted previously, Jackson and Weidman (1958) observed that tea drinking in the UK was a significant factor in bone F levels regardless of drinking water F levels [30].

A study performed in Finland in 1985 found that bone F concentrations were typically 3-fold higher in adult subjects (mean age 60 yrs) who had resided in a fluoridated community for 20 years compared with subjects from nonfluoridated areas [187]. In men, the highest concentration was 2750 ppm. In women, the highest concentration of F in bone was found in a subject with impaired renal function (3890 ppm) [187]. However, it is important to note that tea consumption is uncommon in Finland and the subjects had not ingested fluoridated water throughout their lifetime. Moreover, the study would not have included exposure from fluoridated toothpaste, which only became widely available from the 1980s onwards. Mostafaei et al. (2015) found that the rate of increase in bone fluorine content per year was 3-fold higher for tea drinkers in Canada compared to nontea drinkers [188]. However, it should also be noted that per capita consumption of black tea in NZ is twofold higher than Canada and fourfold higher than Finland [1].

In Switzerland, Boillat et al. (1980) in a study examining occupation exposures to F found that as bone F levels increased, the frequency of joint pain and stiffness increased [189]. This joint pain resulted in disability in some cases. Radiological findings of a higher frequency of ossification of the attachment of ligament, tendon, and muscle to bone were observed in exposed workers compared to workers of similar age and physical activities but without significant occupational exposure to F [189]. The anatomical regions with the highest prevalence of this condition were the heel, hips, spine, forearm, knee, and hand. Up to 100% of the F exposed workers had evidence of skeletal ossification. In this study, the mean age of workers was 61 years and the mean bone F content in the F exposed workers was 5,617 ppm (SD = 2,143 ppm) [189]. Wang et al. (1994) reported similar radiological findings in 127 patients from mainland China (mean age 43 years) with clinically proven skeletal fluorosis [190].

In a study conducted in Inner Mongolia, Li et al. (2009) reported that when drinking water F levels were 0.70 mg/L and the mean F content of tea was 1.81 mg/L, 31% of habitual tea drinkers were found to have skeletal fluorosis [191]. Cao et al. (1996) reported that the consumption of tea infusions with a F concentration of 2.59 mg/L led to an epidemic of fluorosis among Tibetans [192]. The F level in drinking water was 0.11 mg/L and tea consumption was found to contribute >90% of the daily dietary intake of F. Among the 685 adult subjects examined, 31.5% showed signs of skeletal fluorosis. Of these, 8.1% were classified as Grade I, 22.5% as Grade II, and 0.9% as Grade III. Frequency of manifested symptoms included 39.2% with joint pain; 26.1% with lumbago; 16.9% with numbness in the extremities; 4.1% with tetany; and 3.8% with hand and foot rigidity [192]. Wang et al. (1994) found that symptoms most prevalent among adult subjects with skeletal fluorosis were lower back pain, followed by leg pain, joint dysfunction, arm pain, hand tingling, and neck pain [190]. In a study conducted in Thailand, Namkaew and Wiwatanadate (2012) found a significant association between daily F intake and lower back pain and suggested that this could be deemed as the early stage of mild skeletal fluorosis caused by joint or bone degeneration [193].

In the UK, where tea drinking is common, excessive F intake from BT consumption has been found to be associated with chronic arthritis [194]. The author concluded that some cases of pain diagnosed as rheumatism or arthritis may be due to subclinical fluorosis which is not radiologically demonstrable [194]. Similarly, Carnow and Conibear (1981) reported that clinical musculoskeletal effects could occur before skeletal fluorosis becomes apparent radiographically [195]. Consistent with the UK study, numerous other researchers have also found an association between F intake and osteoarthritis [193, 196–203]. Most notably, Ge et al. (2006) in a large-scale study from China found that the rate of osteoarthritis was significantly increased at water F levels of just 1.7 mg/L [196], a concentration within the range of F measurement reported in this study for tea beverages in NZ.

In the USA, researchers in a recent large-scale study of 76,000 US women with ages ranging from 50 to 79 found that women who drink greater than 4 cups of black tea per day have a 78% greater chance of developing rheumatoid arthritis compared to those women who never drink tea. They also reported that women who consume any amount of black tea, even small quantities, per day, have a 40% risk of developing RA [204]. A previous USA study involving predominantly white women aged 55–69 years found that drinking 1–3 cups of tea per day increased the risk of RA, while consuming more than 3 cups per day reduced the risk. The category of tea, however, was not identified [205].

In Sweden, Tettamanti et al. (2011) found an association between tea but not coffee consumption and bladder pain syndrome in women [206]. Unlike smoking, this association was not found to be confounded by genetic factors. Moreover, in Turkey, Seferoglu et al. (2012) found that consumption of more than 4 cups of tea was risk factor for chronification of migraine [207]. Based on Turkish consumption, this would equate to approximately 400 ml of tea [167], which is less than the average intake of BT in NZ.

In addition to bone F levels, blood F levels have been widely used a biomarker of F exposure and indicator of skeletal fluorosis. Xiang et al. (2005) demonstrated that long-term exposure to F resulting in fasting serum F concentrations ranging from 2.5 to 8.0 *μ*mol/L can result in chronic F intoxication and stage I and stage II skeletal fluorosis [208]. Several international studies have confirmed this association. In a study conducted in Turkey, Savas et al. (2001) found that the mean serum F levels in 56 patients with endemic fluorosis were 5.27 *μ*mol/L with knee joint being the most commonly diagnosed abnormality, followed by lower back pain and hip, feet, shoulder, and neck pain [203]. In India, Susheela and Bhatnagar (2002) in a hospital based study diagnosed symptoms of skeletal and nonskeletal fluorosis in patients aged 8 to 60 years of age with serum F levels ranging from 2.1 to 5.0 *μ*mol/L [209]. Symptoms included pain and rigidity in the joints, nonulcer dyspepsia, and gastrointestinal complaints such as nausea, vomiting, pain in the stomach, bloated feeling/gas formation in the stomach, constipation followed by diarrhoea, polyuria and polydipsia, and muscle weakness and fatigue. In another study conducted in India, Harinarayan et al. (2006) documented fluorotoxic metabolic bone disease (FMBD) in 8 patients aged 12 to 58 years with serum F levels ranging from 1.05 to 4.7 *μ*mol/L [210]. Notably, the authors found that FMBD was associated with severe osteomalacia due to mineralization defects predominantly caused by nephrogenic hypocalcemia and hypophosphatemic defects due to renal tubular damage caused by F [210].

In the USA, Whyte et al. (2008) reported a case study of an American woman who developed skeletal fluorosis from excessive tea consumption. Her fasting serum F level was 6.3 *μ*mol/L before the patient stopped drinking instant tea [130]. Four months after discontinuing tea consumption her fasting serum F levels reduced to <1 *μ*mol/L and the patient's pains resolved over several years after she stopped drinking instant tea [130]. Hallanger Johnson et al. (2007) presented case studies of four American women (50 to 67 years of age) who developed F-related bone disease and associated chronic pain from excessive tea consumption [129]. Among the four patients, serum F levels were found to range from 10.2 to 19.0 *μ*mol/L. In all cases reduction of tea consumption was seen to improve symptoms for patients. The authors concluded that F excess should be considered in all patients with a history of excessive tea consumption, especially due to its insidious nature and nonspecific clinical presentation [129].

Isbell and Villareal-Armamento (2010) reported the case of a 45-year-old US male diagnosed with skeletal fluorosis from ingesting 1.87 L of instant tea daily for 10–15 years [211]. Plasma F levels were reported to be 7 *μ*mol/L. Radiographic findings showed that bone mineral density was above the expected range; however, the bones were more brittle and susceptible to fracture [211]. In another USA study, Izuora et al. (2011) reported a case study of a 48-year-old woman with abnormal bone mineral density who suffered severe chronic bone and joint pain and kyphosis (abnormal curvature of the thoracic spine) from excessive consumption of tea over a period of three decades [212]. Painful areas included her elbows, wrists, hips, knees, and ankles. The patient consumed at least 3.8 L of tea daily containing 1.59 mg/L of F, as measured by ion-specific electrode. Serum F levels were 11.5 *μ*mol/L and 24 hr urinary F excretion was 12.5 mg a day. A lateral radiograph of the spine indicated crippling skeletal fluorosis from severe calcification, osteoporosis, and increased bone density. Treatment was provided by avoiding all F in the diet and administering vitamin D and calcium. Six months after cessation of tea drinking, the patient reported nearly complete resolution of her pains [212]. As previously mentioned, Kakumanu and Rao (2013) reported a case study of a 47-year-old American woman who developed severe pain in lower back, arms, legs, and hips and had all her teeth extracted due to brittleness from excessive tea drinking over a period of 17 years. Serum F levels were measured at 23 *μ*mol/L. After discontinuation of tea consumption, her symptoms improved [157].

In France, Hayem et al. (2004) identified 5 patients in their practice in Paris, who developed skeletal fluorosis as a consequence of drinking 0.75 to 2 litres per day of tea over a course of 10 to 25 years [213]. The skeletal fluorosis in these patients was the osteomalacic variety of the disease, in which the bones become softened and weak. As a result of the “F-related osteomalacia,” the patients suffered “spontaneous bone fractures” where their bones fractured without external trauma. Plasma F levels measured two days after the patients' last cup of tea (and thus do not reflect the peak F levels) were 3.9 *μ*mol/L. The authors concluded that heavy and prolonged consumption of tea may be capable of inducing F-related osteomalacia manifesting as unexpected spontaneous bone fractures [213]. Notably, Inkovaara et al. (1975) recommended that ionic plasma F concentrations should not exceed 3 *μ*mol/L to reduce the risk of bone fractures in person over 65 years of age [214].

The findings of an intriguing study published by Kurland et al. (2007) regarding a patient who developed skeletal fluorosis from excessive tooth brushing (six times a day) using fluoridated toothpaste are also worth noting [215]. This case is especially significant as the patient, a 52-year-old white American male, did not reside in a fluoridated community and had no known occupational or environmental exposure to F apart from toothpaste. Analysis of the patient's drinking water sources, including tap water (from a well) and bottled water, showed no detectable F. There was no exposure to mining, welding, or industrial use of hydrofluoric acid, nor exposure to F containing insecticides, niflumic acid, or laundry powders. He stated that he did not swallow toothpaste, used nonfluoridated mouthwash, had semiannual dental visits, but without F treatments, did not drink tea or wine, and had not chewed tobacco, inhaled snuff, or cooked with Teflon pots. Initial observations suggested ankylosing spondylitis until laboratory evaluation of blood and urine and radiological examination confirmed skeletal fluorosis. Serum F levels were found to range from 15 to 18.0 *μ*mol/L. The case uniquely charts the natural history of skeletal fluorosis after F exposure ceases. Within 8 months of documentation of skeletal fluorosis and after avoiding fluoridated dental products, serum F decreased to < 2.5 *μ*mol/L. Within twelve months, some improvement of joint pains and neck stiffness was evident and within two years after diagnosis and apparent elimination of excess F exposure, the patient had complete resolution of his neck immobility and no longer required analgesics [215]. Urinary F, bone mineral density, and bone F levels were also found to decline after removal of F exposure and bone health was found to improve. After a period of 8 years, bone F levels had declined by 36%. However, the authors observed that a period of 32 years would be required before bone F levels would reach the normal range. While this patient did not drink tea or fluoridated water, it nevertheless highlights the contribution of fluoridated toothpaste to F exposure when used excessively.

In the UK, a similar case study was reported by Joshi et al. (2011), concerning a British woman of similar age who presented with a metatarsal fracture and was subsequently diagnosed with skeletal fluorosis. Radiological findings showed marked osteosclerosis of the spine and pelvis and ossification of ligaments. Serum and bone F levels were markedly elevated at 44.2 *μ*mol/L and 15,144 mg/kg, respectively [216]. The patient consumed 1.4 L of black tea daily and used toothpaste excessively, brushing her teeth eight to ten times per day. Daily F intake from tea was estimated at 10.9 mg and 4.2 mg from toothpaste [216]. The striking difference in serum F levels can be accounted for by the contribution of BT.

Previous controlled studies involving children aged 3-4 years have shown that plasma F levels can increase to 3.4 *μ*mol/L after using fluoridated toothpaste containing 1000 ppm F [217]. Recently, Zohoori et al. (2015) measured fasting ionic F levels in nine healthy adults aged 20–35 years (5 men and 4 women) residing in a nonfluoridated community in the UK [218]. The mean baseline ionic plasma F level was reported to be 3.2 *μ*mol/l. During the experiment, subjects were provided with nonfluoride toothpaste to use for a week. After switching to a nonfluoridated toothpaste, the mean baseline plasma F levels were observed to reduce to 0.58 *μ*mol/L. The authors suggested that the high fasting plasma F concentration was due to brushing with a fluoridated toothpaste (1400 ppm F) by participants in the morning before attending the preexperimental session [218].

To put this in context, based on the results obtained from these latter studies and those by Toyota [178], it is evident that tooth brushing with commercial fluoridated toothpaste once a day can provide an exposure greater than that associated with ingesting a cup of BT containing 1.4 mg of fluoride. Taken together, these studies clearly highlight the effect of steady, continuous exposure to F and the impact of cumulative exposures.

To our knowledge, no biomonitoring studies measuring ionic F concentrations have been undertaken in NZ. However, in the USA, Singer and Orphaug (1979) found that mean ionic F in plasma in 264 fasting healthy male and female subjects of different ages who resided in a community with fluoridated water (1 mg/L) ranged from 2.74 *μ*mol/L for subjects aged 10–20 years to 3.89 *μ*mol/L for subjects over 60 years [219]. For persons aged 10 to 60 years and older than 60 years the range of ionic F was 0.53–5.79 and 1.05 to 6.84 *μ*mol/L, respectively [219]. In another USA study, Parkins et al. (1974) examined the plasma of 41 inpatients from 17 to 82 years of age (of whom 36 were currently living in an area with fluoridated water) and found a mean ionic F concentration of 2.46 *μ*mol/L and a range of ionic F from 1.0 to 5.89 *μ*mol/L [220]. Smiley et al. (1991) measured fasting plasma F levels ranging from 3.7 to 4.6 *μ*mol/L. in adults (mean age 45 yrs) residing in fluoridated communities in New York [221]. From this data, it should be pointed out that the general population in fluoridated communities in the USA, have similar or higher exposure to F as workers occupationally exposed to F at a Swedish Aluminium Plant following an eight-hour work shift [222].

Based on this evidence, the F levels in tea products reported in this study and the long history of habitual tea drinking in NZ combined with other additive sources of F exposure including water fluoridation; toothpaste; mouthwash; food; and medication, evidence would strongly suggest that a substantial percentage of adult NZ population would have blood F levels within the established range associated with chronic F intoxication and stage I and stage II skeletal fluorosis [208].

As Krishnamachari (1986) described, a combination of osteosclerosis, osteomalacia, and osteoporosis of varying degrees as well as exostosis formation characterizes the progression of skeletal fluorosis [223]. While F therapy has previously been used extensively in the treatment of osteoporosis, it can result in pathological changes to mineralized bone, biomechanical incompetence, and skeletal fluorosis [224, 225]. While F has been found to increase bone mineral density [226], the increase in bone mass does not necessarily result in an increase in bone strength as the bone is poorly mineralized and exhibits inflammatory foci [227]. Kakei et al. (2007) demonstrated that F intake caused crystal structure defects in calcified hard tissues [228]. Carter and Beaupré (1990) demonstrated that the adverse influences of F on the mineralized bone contribute to losses in trabecular strength which can be caused by the presence of hypomineralized or hypermineralized fluorotic tissue [229]. Kakei et al. (2015) using ovariectomized rats, as an animal model of postmenopausal women, found that F at low concentrations (1.0 mg/l), combined with estrogen deficiency, caused crystal structure defects and accelerated osteoporotic changes in bone. Based on these findings, the authors concluded that F exposure increases bone fragility and may increase the risk of developing osteoporosis [230]. It is acknowledged that women over the age of 50 years become susceptible to osteoporosis because of the loss of estrogen at menopause [231].

It is further acknowledged that oxidative stress and low antioxidant status are risk factors for osteoporosis [232–234]. F decreases oxygen consumption and increases superoxide production in osteoblastic cells and enhances reactive oxygen species (ROS) generation in bone marrow cells [235, 236]. These results indicate that F can damage bone tissue by inhibiting the respiratory chain, increasing the production of superoxide radicals and thus of the other ROS [235]. Human studies have reported that when serum F levels in adults increased from 0.5 to 13 *μ*mol/L the products of lipid peroxidation in blood increase significantly and the antioxidant activity of superoxide dismutase (SOD), catalase (CAT), and glutathione peroxidase (GPx) decreased markedly in F exposed individuals compared to healthy controls [237, 238]. Dlugosz et al. (2009) demonstrated that estrogens protect against F induced toxicity by increasing thiol group levels which act to impair lipid peroxidation and suggested that postmenopausal women could be more sensitive to F induced oxidative stress [239].

Kataraki and Rao (2012) demonstrated that serum F levels and oxidative stress biomarkers increased markedly in woman after menopause compared to nonpregnant women aged 20–40 years residing in the same area [240]. Ishiguro (1991) measured the F content in bones of men and women subjects aged 20 to 93 years and found that the F concentrations in men increased steadily with age; however, in women bone F concentrations increased markedly over the age of 55 years [241]. Notably, Inkovaara et al. (1975) recommended that ionic plasma F concentrations should not exceed 3 *μ*mol/L to reduce the risk of bone fractures in person over 65 years of age [214].

In a large cohort study conducted in Finland, Kurttio et al. (1999) found that higher F intake was associated with increased risk of hip fractures among women aged 50–64 years [242]. Li et al. (2001) examined the association with long-term F exposure and risk of bone fractures in a study group of 8266 male and female Chinese adults aged 50 years and over [243]. Parameters evaluated included F exposure, prevalence of bone fractures, demographics, medical history, physical activity, cigarette smoking, and alcohol consumption. None of the subjects used fluoridated toothpaste or mouthwash. Remarkably, only 13.5% reported tea drinking and the F content in tea brewed tea samples, though not reported, was insignificant [243]. The results confirmed that drinking water was the only major source of F exposure in the study populations. The report found that the prevalence of hip fractures was highest in the group with the highest F intake and lowest in the group with <1 mg/day intake. The risk of hip fracture was twofold higher in subjects with a daily intake of 6.5 mg/day and threefold higher with intake of 14 mg/day compared to the lowest exposure group (<1 mg/day) [243].

In the USA, Sowers et al. (1991) found an association between F intake, osteoporosis, and fracture of the wrist, spine, or hip with increasing F exposure [244]. Several other ecological studies have also suggested an increased risk of hip fracture associated with fluoridation [245–248]. Karagas et al. (1996) found that, for men aged 65 and 90 years living in area with fluoridated water in the USA, the relative rates of fractures of the proximal humerus and distal forearm were significantly increased (by 23% and 16%, resp.) [249]. In another large USA prospective study involving 121,700 women aged 30–55 years of age, Feskanich et al. (1998) found an association between water fluoridation and risk of distal forearm fractures [250]. In a multicentre prospective study conducted in the USA, Phipps et al. (2000) found that postmenopausal women who had lived for more than 20 years in an area with fluoridated water had an increased risk for fractures of the wrist compared to those not exposed [251]. These findings are of concern, considering the reported increase in incidence of distal forearm fracture among USA men and women under 20 years of age in recent decades [252]. Furthermore, Kim et al. (2012) reported that, compared to epidemiologic studies in Japan and European countries, the incidence rates of humerus fractures are substantially higher in the USA; for distal humerus, the peak rate of fractures was highest among children aged 5–9 years [253].

Taken together, there is a substantial body of evidence to support the view that F intake and water fluoridation may be associated with increased risk of osteoporotic fractures, as well as increased risk of bone fractures in the young.

### 4.5. Temporal Changes in Antioxidant Content in Black Tea

In reference to skeletal fluorosis from BT consumption, it is also necessary to examine how the effects of changes in tea manufacturing processes in recent decades may have altered antioxidant activity of BT products. Among the antioxidants in tea, (−)-epigallocatechin-3-gallate (EGCG) is known to be the most potent antioxidant [254]. In general, GT has been found to be superior to BT in terms of health effects, owing to the higher content of EGCG [255, 256]. More importantly, EGCG has been shown to play a crucial role in mitigating the effects of oxidative stress induced damage by F on plasma, bone, and several organs such as the heart, liver, kidney, lung, testes, and brain [257–267]. Carloni et al. (2013) found that, in the manufacture of CTC black tea, a profound loss in total catechin content and antioxidant potential is observed. Most notably, the EGCG content in GT and orthodox BT was almost 50-fold higher than in CTC black tea [268]. Numerous studies have also shown that that the addition of milk to BT, which is the common practice in Western countries, has been found to further reduce the antioxidant potential of BT [269–272]. An unexpected finding is that the addition of milk to GT has been found to enhance the antioxidant capacity of GTs [273, 274].

Based on this evidence, we hypothesise that, in addition to contributing to higher F exposure, the introduction of CTC teabags may also have contributed to profoundly reduced antioxidant intake among BT consumers, thereby increasing the risk of fluorosis related disorders. We further hypothesise that the Western practices of consuming BT with milk may contribute to a state of chronic systemic inflammation with a diminished capacity to compensate for conditions of increased oxidative stress associated with excessive F intake, as well as cumulative exposures to other environmental toxins, including aluminium and pesticide residues such as chlorpyrifos and deltamethrin, which have been shown to have synergistic toxicity with F [275–280].

We also hypothesise that in countries with water fluoridation and history of BT consumption the effects will be disproportionate compared to countries without water fluoridation.

### 4.6. Relevance to NZ Population

The most recent dietary F intake assessment in NZ reported that the mean dietary F intake from all sources, including food, water, beverages, and use of dental products for NZ adults ranged from 1.4 to 2.5 mg/day in communities with fluoridated water and 0.8–1.3 mg/day in nonfluoridated communities [281]. The results of this study show that, in NZ, for individuals residing in communities with fluoridated water supplies, certain popular brands of BT can contribute 4.1–5.9 mg/day for moderate (3-4 mugs per day) and 8.8–14.7 mg/day for heavy tea drinkers (6–10 mugs per day), respectively. Thus, regular consumption of BT can exceed the AI and UL for F without taking cumulative F exposure from other dietary sources including food, water, alcoholic beverages, use of toothpaste or F mouthwash, and tobacco consumption.

It is important to be aware that in NZ and Australia the market share of fluoridated toothpaste is reported to be 98%, which is similar to the US [282]. In comparison, in the 1970 and 1980, fluoridated toothpaste represented 6% and 76% of total toothpaste sales in NZ, respectively [79]. It is noteworthy, as previously discussed, that the prevalence of dental fluorosis in children has increased significantly in NZ since the 1980s [79–81].

It is also important to note that other sources of F such as tobacco are widely available in NZ. In NZ, 26% and 20% of males aged 18–34 and 35–54 years of age, respectively, currently use tobacco. For women, the percentage is 20% and 17%, respectively. Notably, for Maori population, the percentage is much higher with 40% of women 15 years and older daily smokers and 35% of men [283]. Smoking is associated with increased oxidative stress and a compromised antioxidant defence system [284] and tobacco products are known to contain high concentrations of F [98, 285, 286], which suggests synergistic interactions. Several studies have identified that the prevalence of fluorosis and severity of the disease increase with smoking [93–97], which appears to confirm this relationship. Notably, Tu et al. (2010) found that the fluoride levels in blood and urine of regular and occasional smokers were significantly higher than those of nonsmokers [94]. However, as with tea, the F content in tobacco products in NZ or the contribution of tobacco consumption to dietary F intake has not been assessed. Further studies are needed to quantify the F content in NZ tobacco products and synergistic interactions between F and other toxic heavy metals in tea and tobacco.

As previously noted, Warnakulasuriya et al. (2002) found that alcoholic beverages (beers) produced on fluoridated water supplies in the UK and Ireland can be a major contributor to F intake and contribute to metabolic bone disease, particularly among habitual consumers [91]. Further studies are needed to determine the F content in NZ beers and their contribution to dietary F intake among the adult population.

Finally, it is necessary to mention a few health statistics to measure the possible contribution of chronic fluoride exposure to overall health disability burden in NZ. As previously discussed, the most reported adverse health effects of chronic F intoxication are chronic pain and musculoskeletal disorders. There is compelling clinical evidence that musculoskeletal disorders and chronic pain are highly prevalent in NZ and their impact is pervasive. Arthritis is the leading cause of disability in NZ, affecting nearly 1 in 6 people [287]. In 2010, the total financial cost of arthritis in NZ was estimated to be $3.20 billion [287]. Notably, the latest NZ Health survey found that the prevalence of chronic pain and arthritis is increasing. About 620,000 adults (17%) had arthritis with more than half of adults aged 75 years and over (54%) being affected. Moreover, one in five adults were found to be suffering from chronic pain [288]. The prevalence of chronic pain was also found to increase with age affecting 32% of adults aged 65–74 years of age [288]. Swain and Johnson (2014) reported that the most common pain locations among chronic pain sufferers in NZ were lower back (59%), pelvis/abdomen (49%), joints (39%), neck (34%), muscle (31%), and headache (31%) [289]. Notably, Dominick et al. (2011) found that the prevalence of chronic pain was twice as high in New Zealanders of European and Maori ethnicity compared to New Zealanders of Asian ethnicity [290]. This may indicate the preference of GT as the choice of tea beverage among Asians or other genetic factors [291–294].

However, the burden of headaches, migraine, and osteoporosis is also high in NZ. Thomson et al. (1993) reported that the prevalence of bad headaches among adults in NZ was 40.6% with 54.5% of these having the characteristics of bad headache with features symptomatic of migraine [295]. In 2007, it was estimated that over 70,000 men and women were diagnosed with osteoporosis and over 84,000 osteoporotic fractures occurred, costing the NZ economy over $1.15 billion per year. By 2020, it has been reported that NZ can expect nearly 116,000 fractures directly attributable to osteoporosis each year [296].

It is also necessary to mention the burden of chronic kidney disease and diabetes in NZ. Coppell et al. (2013) reported that prevalence of prediabetes among New Zealanders aged 15 years and over was 25.5% with an overall prevalence of diabetes of 7.0% [297]. The prevalence of diagnosed diabetes was found to increase significantly with age affecting 1 in 4 men over 75 and 1 in 8 women, respectively. Prevalence rates among both Maori and Pacific islanders were more than double those of NZ Europeans [297].

Chronic kidney disease and diabetes are characterized by abnormally low glomerular filtration rate [298]. Individuals with kidney disease have decreased ability to excrete F in urine and are at risk of developing fluorosis even at normal recommended limit of F in drinking water [187, 299]. It is well established that that circulating F levels can be 3 to 5 times higher in subjects with renal insufficiency compared to healthy controls [300–302]. Age-associated loss of kidney function has also been recognized for decades [303, 304]. With aging, many subjects exhibit progressive decreases in glomerular filtration rate, which results in increased retention of F causing higher plasma F levels. It has been further suggested that this decreased clearance of F may indicate that elderly people are more susceptible to F toxicity [2, 305]. NZ is currently experiencing a dramatic increase in its older adult population as part of a global aging phenomenon. With this, NZ rapidly aging population (≥65 years of age) is set to increase by 84% between the years 2006 and 2026 [306].

It is important to also note that the NRC (2006) reported that the intake of F for individuals with low iodine status poses significantly higher risk of negative outcomes than for healthy individuals [2]. Recent evidence from a number of studies has indicated the reemergence in iodine deficiency in NZ among both children and adults [307–309].

Taken together, a substantial body of evidence, namely, the history of habitual BT consumption and other dietary sources of F combined with a rapidly aging population, the reemergence in iodine deficiency, prevalence of smoking, and prediabetes and diabetes, suggests that a substantial portion of the adult population in NZ are at high risk of developing skeletal fluorosis.

## 5. Limitations of the Study

No study is without limitations, and it is important to provide some perspective in this context. The objective of this study was to determine the F content in tea infusions and access the range of likely human exposures to F from BT consumption in NZ. However, without biomonitoring of the population, measuring bone, and plasma F levels and without studies examining the direct association between F intake and its possible relationship to chronic inflammatory diseases in NZ, the association between F intake and chronic morbidity cannot reliably be shown to be causal. Therefore, it is not possible to make conclusive statements about the association without further research to confirm the above suggestions. This field of research would therefore benefit from prospective or longitudinal studies assessing associations between F intake, cumulative F exposure, total body burden of F, and the impact of long-term chronic F exposure on the health of older adults.

There are other limitations such as seasonal variation in F content in BT products, pesticide, heavy metal, and polyphenol (EGCG) contents which were not determined. Pesticides residues in tea can be transferred into tea infusion and then consumed by humans [310]. As previously noted, it has also been found that coexposure to F and aluminium or pesticides, such as CPF, results in synergistic interactions and enhanced toxicity [275–280]. Bishnu et al. (2009) reported that CPF pesticide residues have been found in tea at concentrations that may pose health hazards to the consumers [311]. High aluminium concentrations in tea infusions have also been found to result in underrepresentation of F levels in tea infusions when measured by ISE method [312, 313]. However, it should be noted that no funding was provided to undertake this study and the aim of the study was to solely measure the ionic F levels in tea. Moreover, our review of published literature found that the aluminium content in tea infusions is generally never reported when measuring the F content in tea infusions.

A further limitation of this study is that we did not include an exposure assessment for F intake from tea for children or adolescents or discuss other potential nonskeletal effects of F such as endocrine dysfunction, cardiotoxicity, pulmonary toxicity, or neurotoxicity in our review of health impacts, as this would require further research beyond the scope and limitations of this study. Nonetheless, limitations should not be construed as detracting from the message of the paper; rather, they are the type of limitations indicating that further work needs to be done to establish causality. We also opine that this study and our review of published literature represents the most comprehensive summary of the effects of F on the skeletal system and includes solid evidence of a potential causative association between environmental exposure to F and chronic inflammatory disease.

The need to combine clinical and epidemiological strategies to address long-term F accumulation in the adult population may seem obvious, but it is often overlooked in Western countries. The apparent lack of clinical diagnoses of skeletal fluorosis in Western countries may stem from differences in diagnostic practices, which are well established for skeletal fluorosis in Asian countries, due to the fact that skeletal fluorosis is a major public health burden in India and China. Notably, in China, clinicians are also experienced in investigating skeletal fluorosis from tea, due to the culture of tea drinking in China. However, diagnosis of skeletal fluorosis from tea drinking in Western countries is a relatively new phenomenon and largely coincides with the introduction and market dominance of CTC teabags.

Interestingly, in recent years, most studies in Western countries have come from the USA, which has one of the lowest per capita consumption rates of tea internationally, far below that of NZ, Australia, or the RoI, and which also practices water fluoridation as do NZ, Australia, and the RoI. Yet remarkably, we are not aware of any published information documenting skeletal fluorosis from tea in these latter countries.

## 6. Conclusion

The main finding of this study is that tea is an important and underrepresented source of exposure to F in NZ. In our study, we have demonstrated that the potential F intake from BT consumption is much higher than previously recognized in NZ. Moreover, evidence suggests that the culture of tea drinking in NZ contributes significantly to the total body burden of F among New Zealanders. Although specified regulations concerning tea quality are well established internationally, the F content in tea products is not included. Producers of tea products should label the F concentration of their products. Such labelling will allow consumers to make informed decisions and dentists, dental hygienists, and other healthcare professionals to appropriately advise patients regarding F intake and use of fluoride products. Because F toothpastes and other oral healthcare products such as F mouth rinses are widely available, the cumulative exposure from various combinations of F modalities can result in excessive cumulative exposures. In this study, we have presented a potential causal relationship between cumulative F intake, chronic pain, musculoskeletal disorders, arthritic diseases, and osteoporotic fractures and its relevance to NZ. Based on our observations and hypothesis, the substantial amounts of F found in commercial tea brands in this study, and the long history of BT drinking in NZ, combined with other risk factors such as water fluoridation, alcohol consumption, smoking, and diabetes, it is evident that a sizable portion of the NZ population are susceptible to the health risks associated with long-term chronic F intake. Further studies are needed to confirm this association. Taken together, our study illustrates that there is a considerable gap in knowledge and in recognizing and identifying key risk factors that influence policy making decisions surrounding water fluoridation. On the basis of credible evaluations of public health policy, adequate evidence based surveillance systems must be put in place to monitor the variety and sources of environmental exposure to fluoride, including cumulative impacts and synergistic interactions. Such studies need to determine individuals bone and plasma F levels in addition to other molecular biomarkers. In addition, risk factors and behaviours that may be linked to increased susceptibility to fluoride intoxication must also be adequately examined, such as smoking, alcohol consumption, diabetes, nutritional and endocrine disorders, iodine deficiency, gender, age, and genetic susceptibility.

## Figures and Tables

**Table 1:** Tea brands, weight of teabag, and fluoride content in tea infusions.

Sample number	Brand name	Weight of tea bag (g)	Fluoride in tea infusions made with 0.7 mg/L fluoridated drinking water (mg/L)	Fluoride in tea infusion made with deionized water (mg/L)

1	Bell Kenya Bold	3.3	3.9	3.2
2	Bell Original Tea	2.3	3.4	2.7
3	Pams-New World Supermarket Tea	2.4	3.1	2.4
4	Pak-n-Save Supermarket Budget Tea	2.1	2.8	2.2
5	Choysa Extra Strong	2.3	2.7	1.9
6	Twinings English Breakfast Extra Strong	2.7	2.6	1.8
7	Choysa Classic	2.2	1.8	1.3
8	Twinings Classic English Breakfast	2.3	1.7	1.1
9	Woolworths Home Brand Tea	1.9	1.6	0.9
10	Dilmah Organic	2.3	1.5	0.8
11	Chanui Pure Ceylon Tea	2.2	1.4	0.8
12	Twinings Lapsang Souchong Tea	2.3	1.4	0.8
13	Planet Organic Tea	2.2	1.2	0.7
14	Dilmah Premium Ceylon	1.7	1.2	0.6
15	Chanui Irish Breakfast	2.3	1.1	0.5
16	Dilmah English Breakfast Tea	2.0	1.1	0.5
17	Chanui English Breakfast	2.2	0.9	0.4
18	Dilmah Earl Grey Tea	2.2	0.9	0.4

*Note*. Two teabags selected at random from each branded box of tea. Each tea bag was brewed for a period of 5 minutes with 200 mL of water initially at 100°C. After five minutes of extraction, teabags were removed and the infusions were cooled to room temperature (18–20°C). F level of boiled and cooled tap water 0.7 mg/L. F level of deionized water was 0.0 mg/L. The F content of tea infusions made with drinking water is the mean of two samples tested from each brand of tea.

**Table 2:** Summary of fluoride intake per cup for 18 black teabag commodities.

Cups per day	Volume (mls/day)	Tea infusions made with nonfluoridated water (0.00 mg/L) Total F intake		Tea infusions made with fluoridated water (0.70 mg/L) Total F intake		Tea infusions made with fluoridated water (1.0 mg/L) Total F intake	
		Max mg/day	Min mg/day	Max mg/day	Min mg/day	Max mg/day	Min mg/day
1	250	0.8	0.1	1.0	0.2	1.1	0.3
2	500	1.6	0.2	2.0	0.5	2.3	0.8
3	750	2.4	0.3	2.9	0.7	3.2	0.9
4	1000	3.2	0.4	3.9	0.9	4.2	1.2
6	1500	4.8	0.6	5.9	1.4	6.3	1.8
8	2000	6.4	0.8	7.8	1.8	8.4	2.4
10	2500	8.0	1.0	9.8	2.3	10.5	3.0
12	3000	9.6	1.2	11.7	2.7	12.6	3.6
14	3500	11.2	1.4	13.7	3.2	14.7	4.2

In the 28 member countries of the EU, the AI for a healthy adult female is 2.9 mg/day and 3.4 mg per day for a healthy adult male [121]. In the USA, the AI is 3.0 mg and 4 .0 mg per day, respectively [122].

**Table 3:** Summary of fluoride intake per mug for 18 black tea bag commodities.

Mugs per day	Volume (mls/day)	Tea infusions made with nonfluoridated water (0.00 mg/L) Total F intake		Tea infusions made with fluoridated water (0.70 mg/L) Total F intake		Tea infusions made with fluoridated water (1.0 mg/L) Total F intake	
		Max mg/day	Min mg/day	Max mg/day	Min mg/day	Max mg/day	Min mg/day
1	350	1.1	0.1	1.4	0.3	1.5	0.4
2	700	2.2	0.3	2.7	0.6	2.9	0.8
3	1050	3.0	0.4	4.1	0.9	4.4	1.3
4	1400	4.5	0.6	5.5	1.3	5.9	1.7
6	2100	6.7	0.8	8.2	1.9	8.8	2.5
8	2800	9.0	1.1	10.9	2.5	11.8	3.4
10	3500	11.2	1.4	13.7	3.2	14.7	4.2

In the 28 member countries of the EU, the UL for a healthy adult male or female is 7.0 mg/day [123]. In the USA, the UL is 10.0 mg per day [122].

**Table 4:** Summary of fluoride intake from Bell tea products for NZ adults as a percentage of AI and UL based on EU dietary reference.

	350 ml	700 ml	1050 ml	1400 ml	2100 ml	2800 ml	3500 ml
	1 mug	2 mugs	3 mugs	4 mugs	6 mugs	8 mugs	10 mugs
*Bell Kenya Bold*							
F intake (mg/day)	1.4–1.5	2.7–2.9	4.1–4.4	5.5–5.9	8.2–8.8	10.9–11.8	13.7–14.7
*% of AI *							
Adult female	47–51%	94–100%	141–152%^‡^	188–203%	282–303%	377–406	471–506%
Adult male	40–44%	79–85%	120–130%^‡^	161–173%	241–259%	321–345%	401–432%
*% of UL*							
Adult both sexes	20–21%	39–42%	59–63%	78–84%	117–126%^†^	156–168%	195–210%
*Bell Original Tea*							
F intake (mg/day)	1.2–1.3	2.4–2.6	3.6–3.9	4.8–5.2	7.1–7.8	9.5–10.4	11.9–13.0
*% of AI *							
Adult female	41–45%	82–89%	123–134%^‡^	164–179%	246–268%	328–357%	410–447%
Adult male	35–38%	70–76%	105–114%^‡^	140–152%	210–229%	280–305%	350–381%
*% of UL *							
Adult both sexes	17–19%	34–37%	51–56%	68–74%	102–111%^†^	136–148%	170–185%

^‡^Excluding all other sources of dietary F intake, the consumption of more than 2 mugs of Bell tea products will exceed the AI for healthy adult males and females. ^†^Consumption of 6 mugs will exceed the UL.

## Abbreviations

AI: Adequate Intake
BT: Black tea
CPF: Chlorpyrifos
DRVs: Dietary reference values
EU: European Union
EFSA: European Food Safety Authority
F: Fluoride
GT: Green tea
NRC: National Research Council
NZ: New Zealand
RoI: Republic of Ireland
RME: Reasonable maximum exposure
SCHER: Scientific Committee on Health and Environmental Risks
TISAB: Total ionic strength adjustment buffer
UK: United Kingdom
UL: Tolerable Upper Intake Level
USA: United States of America
WHO: World Health Organization.
